# Influence of Occult Hepatitis B Infection on Blood Transfusion Safety and Its Countermeasures

**DOI:** 10.3390/pathogens14040301

**Published:** 2025-03-21

**Authors:** Meng Yi, Shuchang Dai, Lin Fang, Bo Pan, Bin Fan, Yiming Pan, Zhong Liu

**Affiliations:** 1 Institute of Blood Transfusion, Chinese Academy of Medical Sciences, Peking Union Medical College, Chengdu 610052, China; yimeng@student.pumc.edu.cn (M.Y.); b2024019008@student.pumc.edu.cn (S.D.); 2445010601@stu.ahmu.edu.cn (L.F.); sxspanbo@student.pumc.edu.cn (B.P.); sxsfanbin@student.pumc.edu.cn (B.F.); ympan@student.pumc.edu.cn (Y.P.); 2 China Key Laboratory of Transfusion Adverse Reactions, Chinese Academy of Medical Sciences, Chengdu 610052, China; 3 School of Public Health, Anhui Medical University, Hefei 230032, China; 4 School of Population Medicine and Public Health, Chinese Academy of Medical Sciences, Peking Union Medical College, Beijing 100730, China

**Keywords:** occult hepatitis B infection, safety of blood transfusion, HBV, voluntary blood donation

## Abstract

Occult hepatitis B infection (OBI) is a serious public health issue. Although a number of effective hepatitis B vaccines are available, hepatitis B still poses a threat to global public health. Patients with OBI are usually asymptomatic, but there may be active HBV DNA present in their blood, leading to the risk of virus transmission during blood transfusions or organ transplantation, constituting a hazard to the health of recipients and increasing the risk of liver cirrhosis and liver cancer. Although China has progressed in the development of blood-screening technology, OBI is still a significant hidden danger to blood transfusion safety. Therefore, in blood screening and blood transfusion, strengthening the monitoring and management of OBI is crucial to ensure blood safety and protect public health.

## 1. Introduction

Although an effective vaccine against hepatitis B virus (HBV) infection has been available for more than 30 years, hepatitis B resulting from HBV infection remains a significant worldwide public health issue [1]. Research indicates that around 25% of cases of chronic hepatitis B (CHB) may progress to cirrhosis or hepatocellular cancer [2,3]. As per the World Health Organization’s (WHO) statistics for 2021, approximately 296 million individuals worldwide suffered from chronic infection with the hepatitis B surface antigen (HBsAg) in 2019, resulting in around 820,000 annual fatalities due to HBV-related liver fibrosis and hepatocellular carcinoma (HCC), with Africa and the Western Pacific exhibiting the highest prevalence rates [4,5].

Transfusion-transmitted HBV continues to be a serious public health concern, and blood-borne transmission is still a major route by which HBV is contracted worldwide. The WHO 2023 report states that the prevalence rates of transfusion-associated HBV infection are 0.02% in high-income countries and 2.81% in low-income countries [6]. Despite the WHO’s recommendations for universal HBV testing of all donated blood, HBV persists as a major threat to blood safety, particularly in resource-limited settings where gaps in the implementation of screening protocols and diagnostic sensitivity exacerbate transmission risks.

Occult HBV infection (OBI) is described as the presence of replication-competent HBV DNA in the blood and/or liver of individuals who test negative for HBsAg using the existing techniques [7]. Despite significant reductions in transfusion-transmitted HBV risks through current blood-screening measures, OBI persists as the predominant residual threat due to its HBsAg-negative profile. A meta-analysis estimates the global prevalence of OBI to be 0.82% among the general population, with marked disparities across nations. Countries with a high Sociodemographic Index (SDI) exhibit a prevalence of 0.36%, contrasting sharply with 3.094% in low-SDI countries [8]. A systematic retrospective analysis indicates that the rate of OBI among blood donors mirrors the overall prevalence of HBV at 0.98% (0.44–1.72) in high-prevalence countries, 0.12% (0.04–0.23) in medium-prevalence countries, and 0.06% (0.00–0.26) in low-prevalence countries (I^2^ = 97·72%; *p*-value for heterogeneity between groups was 0.0012) [9].

Based on the serum anti-HBc status, OBI is often categorized into serologically positive OBI and serologically negative OBI [10]. OBI usually presents as an asymptomatic infection that cannot be identified by current serological testing methods [11]. Research indicates that HBV DNA present in the bloodstream of patients with OBI is detectable only occasionally, with concentrations typically below 200 IU/mL (about 1000 copies/mL) [12]. A defining characteristic of OBI is the persistence of minute quantities of covalently closed circular DNA (cccDNA) of HBV within hepatocytes, in the context of suppressed immune function in the host [13]. In situations where the host’s immune system becomes impaired, HBV may resume replicative processes, thereby increasing the likelihood of developing cirrhosis and HCC within the host organism. Simultaneously, it may facilitate the inadvertent transmission of HBV, as in the cases of organ donation and blood transfusion [14].Blood screening is essential in mitigating the transmission of infectious diseases through blood transfusions. The advancements in blood-screening techniques, blood safety monitoring, and pathogen inactivation technologies have significantly enhanced blood safety. Nonetheless, existing detection tools render OBI a significant concern in blood safety.

To examine the status of the research on OBI and its impact on blood transfusion safety, we employed key terms such as “occult hepatitis B infection”, “blood transfusion”, “occult HBV infection”, “pathogenic mechanism”, and “diagnostic methods“ to search for pertinent studies in the MEDLINE, Web of Science, and other databases from 2015 to 2024, while also incorporating significant early research into the analysis. We assessed 53 studies by filtering the retrieved data, excluding those that did not clearly specify the sample size and positivity rate, including the most recent research from both high-prevalence regions (Africa, India, etc.) and low-prevalence regions (the United States, Canada, etc.). The recent advancements in the pathophysiology and diagnostic techniques related to OBI were also examined. This work evaluates and synthesizes the virological and epidemiological attributes of OBI, the current state of research on detection methodologies, and experiences in the management of blood donations from patients with OBI while underscoring the risks posed by OBI to blood transfusion safety.

## 2. Virology and Epidemiology of OBI

The enduring stability and efficient replication of cccDNA in hepatocytes have consistently been regarded as the primary molecular foundation of OBI [15]. It is widely recognized that there are two distinct equilibrium states in HBV infection within the human body [16]: 1—the equilibrium between viral replication within hepatocytes and the clearance of virus particles from the circulatory system; and 2—the harmonious state between the production and degradation processes of the cccDNA pool. While the host’s immunological response and pharmacological intervention primarily disrupt the first equilibrium, they exert less influence on the latter [17,18]. The half-life of cccDNA in vivo may extend up to 26 months, depending on both the host’s immunological states and treatment regimen, whereas HBV-infected cells may persist for as long as 100 days [19,20,21]. Due to the inadequacy of host immunological regulation and the constraints of antiviral pharmacotherapy, current antiviral treatments cannot entirely inhibit the creation of viral DNA in hepatic cells [22,23]. Even when HBsAg-negative for 30 years, HBV DNA may still be detectable in the liver of some people [24,25].As this demonstrates, following HBV infection, the cccDNA or OBI status in the liver may persist for an extended duration, potentially for a lifetime.

Recent research has demonstrated that the epigenetic regulation of HBV cccDNA is significant in the etiology of OBI. Table 1 illustrates that epigenetic modifications occurring after translation, encompassing methylation of both histones and non-histone proteins, interactions with host cellular transcription factors, as well as ubiquitination processes exert significant impacts on the transcriptional activity and replication dynamics of cccDNA. SIRT3 functions as a crucial host determinant, suppressing replication by impairing the interaction between RNA polymerase II and cccDNA [26]. Nonetheless, SIRT1, the TIP60 complex, HDAC11, and HAT1 may promote OBI activation [27]. SIRT1 is a class III histone deacetylase that facilitates HBV replication in hepatocytes [27]. The TIP60 complex further binds to the HBV promoter, inhibiting HBV transcription induced by the pre-core/core promoter [28]. Specific enzymes modulate the replication and transcription of HBV cccDNA by modulating the histone methylation state, including LSD1, PRMT5, and NIRF. Research indicates that a reduction in LSD1 levels will restrict the expression of viral genes. This discovery pertains to the methylation of the transcription repressor H3K9 and the decrease in activation indicators H3 and H3K4 on the viral promoter [29]. PRMT5 negatively regulates HBV replication by inhibiting cccDNA transcription and disrupting the development of pre-genomic RNA [30]. Recent studies indicate that the intricate modulation of HBV cccDNA in patients is the key driver of OBI [13,31,32].

Moreover, HBV in the OBI population often exhibits mutations. A study sequenced 104 OBI plasma samples and 524 HBsAg-positive samples from 29 blood centers, investigating the high-frequency mutation of the S protein transmembrane domain (TMD) among the OBI community [38]. The TMD mutation of the S protein may produce OBI by decreasing HBsAg secretion, altering HBsAg hydrophobicity, and facilitating HBsAg accumulation in the cell membrane. Current detection techniques may be inadequate for identifying the HBsAg mutant protein, potentially resulting in missed detections [38]. For instance, a Japanese study reported a high OBI prevalence in HIV-infected patients, identifying common mutations in the S protein TMD and core protein. These mutations may help cause OBI by changing the viral antigenicity and preventing immune recognition [39]. Another study emphasized how key protein mutations contribute to the development of OBI, making its diagnosis and treatment even more challenging [40]. The need for prolonged antiviral therapy in individuals chronically infected with HBV contributes to the formation of immunological complexes between free HBsAg in peripheral blood and anti-HBs, a significant factor in the ineffectiveness of current HBsAg detection techniques [41]. Consequently, the protracted nature and complexity of HBV infection, along with insufficient comprehension of the pathogenic mechanisms and restricted detection tools, have led OBI to be identified as a significant concern with regard to blood safety.

Recent domestic research indicates that the frequency of OBI significantly differs among patients with varying HBV serological statuses [9,42,43]: the detection rate of serum HBV DNA is 15.61% in anti-HBe (+)/anti-HBc (+) patients. In comparison, it is 1.87% in anti-HBs (+)/anti-HBc (+) patients [43]. The findings indicate that the serological detection rates of anti-HBs and OBI show a negative correlation in Asia but a positive correlation in Africa, consistent with the effects of anti-HBs and anti-HBe. This is attributable to anti-HBs acting as a protective antibody, whereas anti-HBe signifies a robust immune response, indicating that the individual remains in the immune clearance or recovery phase [44,45]. A study by Candotti et al. suggests that even with a sensitive nucleic acid detection technique (detection limit: 3.4 IU/mL) for three consecutive assessments, blood donors testing negative for HBsAg and HBV DNA are still susceptible to HBV transmission through blood transfusions [46].

There are notable disparities in OBI frequency among blood donors across various groups and countries. Table 2 summarizes the relevant studies on OBI among blood donors from 2015 to date. The prevalence of OBI in affluent nations, such as the United States and Switzerland, is around 0.05%. The prevalence of OBI in poor regions, such as India, Nigeria, and Africa, is often elevated due to factors such as inadequate medical infrastructure and a high incidence of HBV. Furthermore, the true prevalence may be underestimated due to study limitations. Since 2015, China has increasingly focused on the prevalence rate of OBI among blood donors. Table 3 indicates that the frequency of OBI among blood donors in China has shown a declining trend since 2015, approaching the figures seen in industrialized nations. The prevalence rate varies significantly across various areas of China, with a more significant incidence of OBI in the southern regions (Shenzhen, Guangzhou, etc.) compared to the northern areas (Beijing, Liaoning, etc.).

The transfusion of OBI-derived blood may further elevate supplementary dangers. The recipients’ immunological function and underlying disorders may reactivate OBI, particularly in immunosuppressed individuals [86]. Additionally, the research indicates that OBI is strongly associated with HBV-related cirrhosis and HCC, and the interplay between HBV DNA and HCC-related genes may facilitate the progression of HCC [87]. Although no direct research indicates that transfusions of OBI-derived blood present an elevated risk to recipients with fundamental liver diseases, studies have suggested that such patients may struggle to eradicate low-level HBV infections due to their compromised immune function or abnormal liver function [88]. Concurrently, HBV DNA in OBI is inclined to integrate into hepatocyte DNA, potentially exacerbating the illness’s fundamental pathological alterations and intricacy, elevating the hepatic burden, and hastening the disease progression [89]. Consequently, it is essential to focus more on the dangers associated with OBI while administering blood transfusions to patients with underlying liver conditions.

## 3. Detection Method of OBI and Blood Transfusion Safety

The definitive and indispensable criterion for establishing a diagnosis of OBI involves the identification of HBV DNA with replicative capacity in the liver tissues or bloodstreams of patients who are HBsAg-negative [7]. The definitive diagnostic criterion is the detection of HBV DNA in liver tissue; nevertheless, its implementation remains challenging. The conventional approach for diagnosing OBI is the detection of HBV DNA in the blood, while detecting anti-HBc in the blood is an alternative method.

In developed areas such as the United States and European nations, NAT has emerged as the standard for blood screening due to its increased sensitivity and specificity, with accuracy maintained by frequent updates of detection guidelines and stringent quality control of equipment [90]. A combination screening technique including HBsAg, anti-HBc, and anti-HBs was presented in response to the need for blood screening in impoverished regions [91]. This technique has superior sensitivity and cost-effectiveness compared to costly ID-NAT and Minipool NAT, which exhibit a significant false-negative rate, making it a more viable option.

Over the last several decades, the HBV screening protocol for blood donors in China has evolved through three phases [92,93,94]: The first phase encompassed the era of voluntary blood donation before the enactment of China’s Blood Donation Law in 1998. Before blood donation, HBsAg was identified in the laboratory using reverse hemagglutination. Between 1990 and 1998, this technique was gradually replaced by enzyme-linked immunosorbent assay (ELISA). Individuals who receive a negative test result could donate without further testing. In the second phase, spanning from 1998 to 2015, a comprehensive campaign to encourage voluntary blood donation was implemented nationwide. This initiative was complemented by a comprehensive assessment, which encompassed a questionnaire probing into risky behaviors and substance abuse, a thorough physical examination, as well as pre- and post-donation dual screening for HBsAg.

Since 2010, several nucleic acid testing (NAT) technologies have been assessed in many pilot blood facilities in China. The third phase spans from 2015 to the present. Prospective blood donors often undergo swift HBsAg screening before donation, while the blood obtained from eligible donors is subsequently tested using two distinct ELISA methods, and NAT also analyzes HBV DNA.

Despite the prevalent use of NAT and serology for HBV detection, OBI remains a significant risk [95,96]. The challenge of OBI blood screening is excluding samples with low viral loads. The nucleic acid test findings of low viral load samples have a Poisson distribution, indicating that “detected” samples may provide negative results upon re-examination, complicating the differentiation between false negatives and true negatives [97]. Moreover, samples exhibiting a very low viral load present difficulties for identification and may result in transfusion-related HBV infection owing to the transfusion of such blood. Candotti et al. documented a case of HBV infection in Slovenia resulting from the transfusion of blood products collected from OBI blood donors with minimal viral loads. The study investigated three individuals who were recurrent blood donors, having tested negative for HBsAg and exhibited negative results for HBV DNA through NAT. Nine recipients developed confirmed HBV infection after the transfusion of blood components, with seven infections originating from fresh frozen plasma and two from red blood cells containing plasma. The analysis of viral sequences indicated that the sequences from five receivers exhibited over 99% congruence with those of the donors. This research demonstrates that the minimal threshold of HBV infection may be revised from 20 IU/mL to around 3.0 IU/mL of HBV DNA. The lower detection limit of NAT sensitivity required to avert HBV transmission by blood transfusion is 0.15 IU/mL [46]. Consequently, despite the sensitivity of current HBV screening techniques and the safety of blood recipients, OBI remains a significant risk to the safety of blood transfusions.

Recent research on novel biomarkers for OBI diagnosis includes serum HBV RNA, a promising candidate [89]. Serum HBV RNA mainly consists of pgRNA (pre-genomic RNA), regarded as direct evidence of the transcriptional activity of HBV cccDNA. Compared to other biomarkers like HBV DNA and HBsAg, it may provide a more direct and effective means of identifying viral activity in individuals exhibiting viral inhibition (particularly OBI) post-treatment. Nonetheless, the molecular attributes of serum HBV RNA remain poorly understood, and the absence of standardized detection techniques results in restricted comparability across various study findings. The connection between serum HBV RNA and cccDNA significantly varies between studies, perhaps impacted by variables such as patient characteristics and detection methodologies. The clinical use of serum HBV RNA, particularly in comparison to the current indicators, requires more investigation [98].

## 4. Strategies to Mitigate the Risk of OBI During Blood Transfusions

Summarizing, comparing, and deriving insights from advanced experiences would enhance the management efficacy of OBI blood transfusion and assure transfusion safety. Through extensive practice, nations globally have amassed considerable expertise in the administration of blood donation for OBI patients, primarily in the following areas.

### 4.1. Enhance the Sensitivity and Specificity of Detection

In China and most nations in Europe and America, NAT is used as a blood-screening technique, significantly decreasing the risk of OBI [99]. The blood management organization will consistently revise testing guidelines and rigorously oversee the quality of testing equipment to guarantee the use of the newest technologies and standards as well as the reliability of testing outcomes [79,80]. Simultaneously, nations are augmenting investments in researching and developing more precise and effective detection methodologies. Research indicates that digital PCR (ddPCR) has greater sensitivity and accuracy than real-time fluorescence quantitative PCR (qPCR) in identifying low viral loads [100]. The use of ddPCR technology is anticipated to enhance the precision and safety of blood screening, hence offering a more dependable assurance for blood safety management [101]. Nowadays, several ddPCR platforms have been commercialized, such as Raindrop Digital PCR, the Bio-Rad QX200 Droplet Digital System, and the Naica System. However, to complete an assay using these platforms, most require the synergy of several pieces of equipment, such as a droplet generator, droplet reader, and PCR thermocycler [102,103]. In addition, due to the high price of detection equipment and low detection throughput, ddPCR technology has not yet been promoted to clinical applications and is still in the scientific research stage [104]. Table 4 shows the advantages and limitations of the three assays, ELISA, NAT, and ddPCR.

### 4.2. Strict Blood Donor Screening and Feedback System

A thorough health exam is required for all American blood donors. This rigorous regulation reduces the OBI risk [107]. China’s blood donation policy is also improving. However, regional screening standards vary owing to economic and resource restrictions. European and American blood monitoring and feedback systems are excellent, allowing them to detect and fix blood donation issues promptly. Blood donors will be traced after OBI cases are found, and screening procedures will be revised to ensure blood safety. China has improved data monitoring recently. However, countrywide data integration and real-time monitoring require improvement, and most blood donor management systems lack complete information exchange, which delays OBI case detection and feedback [108].

### 4.3. Prevention Through Vaccination

One of the main methods for preventing HBV infection is active immunization. Among vaccinated blood donors, the incidence of HBV has been significantly reduced [109]. Nevertheless, OBI remains an undeniable residual risk [110]. In the United States, a small proportion of vaccinated donors exhibit HBV DNA positivity despite testing negative for HBsAg, demonstrating the persistence of OBI even in regions with a high vaccination coverage [111]. Cai et al. identified OBI in a subset of vaccinated donors, further underscoring this concern [43]. These findings indicate that while hepatitis B vaccination has achieved remarkable success in reducing HBV prevalence, OBI may still occur, signifying that vaccination is not completely effective. Sustained surveillance and enhanced screening technologies remain critical safeguards against transfusion transmission. It must be emphasized that although vaccination-based prevention strategies do not entirely eliminate OBI, they remain essential given the current absence of superior alternatives. Vaccination continues to be a necessary intervention to mitigate the population-level HBV burden while we await advanced preventive measures.

### 4.4. Engage in Public Education Initiatives

China is likewise behind wealthy nations in public education. In the United States and other European countries, public education often encompasses fundamental understanding of the transmission pathways, prevention strategies, and possible health hazards associated with HBV. Community lectures, online courses, publications, and other multi-channel educational initiatives provide extensive information to the public. Moreover, tailored educational programs are implemented for specific demographics (such as high-risk populations) to improve their comprehension of OBI. Public health organizations in several countries will collaborate with NGOs to implement focused health promotion initiatives [112]. In China, the curriculum of public education is progressively being enhanced. However, it remains comparatively limited. While several cities have conducted awareness campaigns for hepatitis B, most of the content emphasizes fundamental understanding and preventative strategies, lacking comprehensive details about OBI and associated risks. Furthermore, owing to disparities in regional development, public educational resources and information access avenues in rural locations remain limited. The interaction is minimal, and most educational initiatives remain one-dimensional, characterized by a lack of substantive engagement with the public.

## 5. Conclusions and Future Perspectives

Despite the ongoing efforts to mitigate OBI blood transfusion risks in China, numerous urgent issues persist, including the constraints of screening technology, inadequate public awareness, uneven regional development, and incomplete information integration and monitoring systems. Consequently, to address the issues posed by OBI, China’s future developmental trajectory may begin with the following aspects: 1. Enhance the sensitivity and accuracy of current detection techniques (such as NAT technology) while simultaneously developing and implementing new technologies. 2. Enhance the quality of public education and awareness. 3. Enhance the dissemination of information about HBV and OBI through multiple channels (such as online platforms, community initiatives, and educational institutions), mainly targeting high-risk populations, and develop tailored educational strategies to improve their self-screening awareness. 4. Enhance regional collaboration and resource consolidation. In particular, enhancing resource sharing and collaboration across various areas is essential. Establishing a regional blood safety network may facilitate data and experience sharing, thereby enhancing overall management efficacy.

This study has certain limitations: Despite the increasing focus on the detection, management, and treatment of occult hepatitis B infection in various countries, regional prevalence disparities and the efficacy of detection technology continue to pose significant challenges to mitigating the risk of OBI to blood transfusion safety. The sample sizes from various investigations remain limited, perhaps failing to accurately represent the actual frequencies of OBI among blood donors in the regions sampled. Furthermore, study findings in the same area across various periods indicate a shifting trend. It is crucial to consistently analyze an extensive sample size to ascertain the prevalence rate of OBI among blood donors. However, it is challenging to accurately study the situation, and the analysis of the available data in this work may be skewed relative to reality owing to the paucity of research in Africa and the small sample sizes of the research that is currently available. Enhancing studies in undeveloped regions like Africa will significantly contribute to a thorough understanding of the impact of OBI on global blood transfusion safety. Consequently, with technological advancements and heightened public health awareness, the risk that OBI presents to blood transfusion safety is anticipated to diminish progressively.

## Figures and Tables

**Table 1:** Epigenetics of cccDNA in OBI.

Type	Target	HBV cccDNA	HBV DNA	HBsAg	Reference
Histone	NIRF	−	−	−	[33]
	LSD1	−	−	−	[29]
	PRMT5	−	−	−	[30]
Regulatory protein	HBx	+	+	−	[34]
	HBx mutations	+	−	−	[35]
Transcription factor regulation	ZHX2	+	−	−	[36]
	RXRα	+	+	−	[37]
Acetylation	SIRT3	+	−	−	[26]
	SIRT1	−	+	−	[27]
	TIP60	−	−	−	[28]

“+” positive correlation; “−” negative correlation; NIRF Np95/ICBP90-like RING finger protein; LSD1 histone lysine demethylase-1; PRMT5 protein arginine methyltransferase 5; HBx HBV regulatory protein X; ZHX2 zinc fingers and homeoboxes 2; RXRα retinoic acid X receptor α; SIRT3 silent mating type information regulation 2 homolog 3; SIRT1 silent mating-type information regulation 2 homolog 1; TIP60 histone acetyltransferase Tip60.

**Table 2:** Characteristics of OBI prevalence in international blood donors.

Fist Author	Publication Year	Location	OBI Infection	Sample Size	OBI Prevalence
Asim M [47]	2010	New Delhi, India	31	2175	1.42529%
Yuen MF [48]	2011	Hong Kong, China	67	217,595	0.03079%
Maria GM [49]	2011	Mexico	24	20,328	0.11806%
Lovrantova E [50]	2011	Slovak	15	65,010	0.02307%
Kim MJ [51]	2012	South Korea	23	149,471	0.01538%
El-Ghitany [52]	2013	Alexandria, Egypt	21	508	4.13385%
Zeinab NS [53]	2013	Egypt	52	3167	1.64193%
Alizadeh Z [54]	2014	Iran	2	500	0.4%
Muselmani W [55]	2014	Syria	5	3896	0.12834%
Niazi SK [56]	2015	Northern Pakistan	719	56,772	0.01266%
Oluyinka O [57]	2015	Nigerian	72	429	16.78322%
Graba P [58]	2015	Poland	2	421,447	0.00047%
Belen P [59]	2016	Córdoba, Argentina	4	70,102	0.0057%
Cinzia SK [60]	2016	South India	4	24,338	0.01644%
Mardian Y [61]	2017	Indonesia	17	456	3.72807%
Athira KP [62]	2018	India	52	1102	4.71869%
Dodd RY [63]	2018	USA	433	22,370,271	0.00194%
Alzahrani FM [64]	2019	Eastern Saudi Arabia	12	22,842	0.05253%
Andrea Z [65]	2022	Switzerland	7	11,432	0.06123%
Sheila OB [66]	2023	Canada	61	1,401,603	0.00435%
Ondigui JLN [67]	2022	Africa	2760	18,579	14.8%

**Table 3:** Characteristics of OBI prevalence in Chinese blood donors.

Fist Author	Publication Year	Location	OBI Infection	Sample Size	OBI Prevalence
Liu Y [68]	2010	Nanjing, China	5	2972	0.16824%
Lie YS [69]	2011	Shaoxing, China	8	8692	0.09204%
Dong J [70]	2014	Hangzhou, China	86	178,447	0.04819%
Zhou S [71]	2015	Shenzhen, China	99	310,167	0.03192%
Lin H [72]	2016	Jiangsu, China	81	157,119	0.05155%
Liu C [73]	2016	Beijing, China	13551	20,084,187	0.06747%
Liao H [74]	2017	Baoji, China	60	110,843	0.05413%
Ye XL [75]	2017	Shenzhen, China	6	1134	0.5291%
Lin H [76]	2017	Jiangsu, China	80	134,495	0.05948%
Tang X [77]	2018	Guangzhou, China	10	14,937	0.06695%
Ye XL [78]	2019	Shenzhen, China	162	123,280	0.1314%
Ye XL [79]	2022	Heyuan, China	70	44,592	0.15698%
Chen JF [80]	2023	Shandong, China	69	220,445	0.0313%
Deng XL [81]	2023	Liaoning, China	451	869,633	0.05186%
Wang R [82]	2024	Beijing, China	76	212,134	0.03583%
Mo YP [83]	2024	Huzhou, China	31	44,256	0.07004%
Ni XW [84]	2017	Jiaxing, China	47	52,698	0.089%
Jing YY [85]	2015	Xi’an	171	263,501	0.06%

**Table 4:** Comparison of three detection methods.

Technique	ELISA	NAT	ddPCR
Target	Antigen or antibody	Nucleic acid	Nucleic acid
Theory	Antigen–antibody reaction	Nucleic acid amplification (e.g., PCR, RT-PCR) to detect DNA or RNA of pathogens	The samples were divided into many independent reaction chambers for PCR amplification and fluorescence signal detection, and the copy number of the target molecule was calculated based on the Poisson distribution
Sensitivity	pg/mL	The sensitivity of HBV nucleic acid detection was 100% [105]	It can detect rare mutations or single-copy nucleic acids as low as 0.001% [104]
Specificity	Low specificity and possible cross-reactivity	High	High
Time of detection	1.5–4 h	1.5–2.5 h	4–6 h
Detection flux	High throughput (96-well plate)	Medium (requires tube-by-tube amplification)	Low (droplet generation efficiency-dependent) [106]
Cost	Low	High	Highest (expensive equipment, high cost of reagents)
Generalizability	Suitable for mass screening, especially in resource-limited settings	Dependent on professional and laboratory conditions	Specialized training and data interpretation are required
For OBI	Combined test (HBsAg + anti-HBc + anti-HBs) is used for initial screening in resource-limited areas, but window period exists	Shortening the window period and supporting typing is the gold standard for diagnosis	No need for standard curve, accurate calculation of viral copy number, currently applicable to scientific research, accurate monitoring of OBI viral loads

ELISA Enzyme-linked immunosorbent assay; NAT Nucleic acid testing; ddPCR Droplet digital PCR; PCR Polymerase chain reaction; RT-PCR Reverse transcription–polymerase chain reaction; HBV Hepatitis B virus; HBsAg Hepatitis B virus surface antigen; anti-HBc Anti-hepatitis B core antigen; anti-HBs Anti-hepatitis B surface antigen; OBI Occult hepatitis B infection.

## Data Availability

Data will be made available on request.
